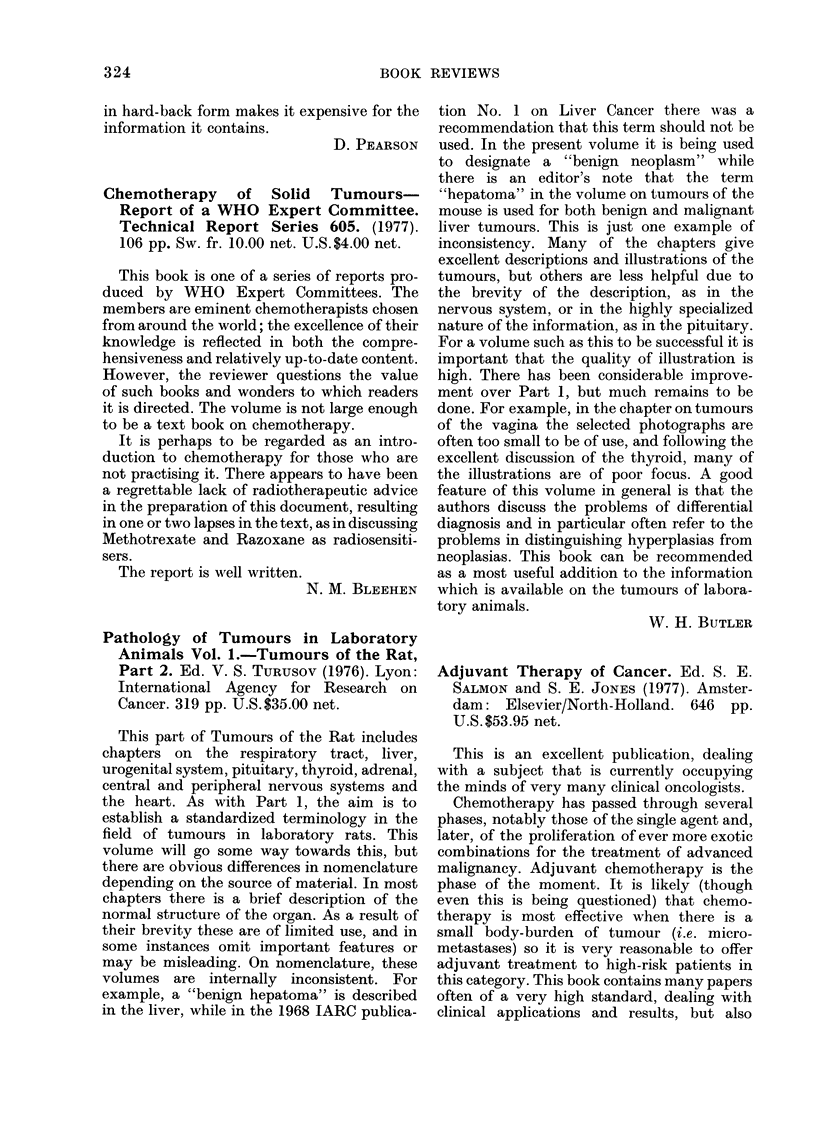# Chemotherapy of Solid Tumours—Report of a WHO Expert Committee. Technical Report Series 605

**Published:** 1978-02

**Authors:** N. M. Bleehen


**Chemotherapy of Solid Tumours—Report of a WHO Expert Committee. Technical Report Series 605.** (1977). 106 pp. Sw. fr. 10.00 net. U.S.$4.00 net.

This book is one of a series of reports produced by WHO Expert Committees. The members are eminent chemotherapists chosen from around the world; the excellence of their knowledge is reflected in both the comprehensiveness and relatively up-to-date content. However, the reviewer questions the value of such books and wonders to which readers it is directed. The volume is not large enough to be a text book on chemotherapy.

It is perhaps to be regarded as an introduction to chemotherapy for those who are not practising it. There appears to have been a regrettable lack of radiotherapeutic advice in the preparation of this document, resulting in one or two lapses in the text, as in discussing Methotrexate and Razoxane as radiosensitisers.

The report is well written.

N. M. Bleehen